# Supplementary material for: A Hormetic Spatiotemporal Photosystem II Response Mechanism of Salvia to Excess Zinc Exposure
Source: Int J Mol Sci. 2022 Sep 23;23(19):11232. doi: 10.3390/ijms231911232 (PMC9569477; doi:10.3390/ijms231911232)
Supplement: Supplementary file 1 [file ijms-23-11232-s001.zip › ijms-1867152-supplementary.pdf]

A Hormetic Spatiotemporal Photosystem II Response Mechanism of Salvia to Excess Zinc Exposure

Michael Moustakas *, Anelia Dobrikova, Ilektra Sperdouli, Anetta Hanć, Ioannis-Dimosthenis S. Adamakis, Julietta Moustaka and Emilia Apostolova

Table S1. Operating conditions for laser ablation inductively coupled plasma mass spectrometry (LA-ICP-MS) system

Laser Ablation	Operating Conditions
Instrument	CETAC LSX-500, Nd-YAG
Wavelength [nm]	266
Ablation frequency [Hz]	10
Spot size [μm]	100
Laser energy [mJ]	5.4
Scan rate [μm/s]	80
Distance between scan lines [μm]	20
Scan method	Mapping 2D; scanning
**ICP-MS**	
Instrument	PE Sciex ELAN 6100 DRC II
Nebulizer gas flow [L/min]	1.1
Auxiliary gas flow [L/min]	1.2
Plasma gas flow [L/min]	16
RF Power [W]	1350
Lens setting	Autolens calibrated
Detector mode	Dual (pulse counting and analog mode)
Measured mass to charge ratios	Mg (m/z 26); Zn (m/z 66); Ca (m/z 43); C (m/z 13)
Sweeps	1

Table S2. The measured chlorophyll fluorescence parameters with their definitions

Parameter	Definition	Calculation
F_v/F_m	Maximum efficiency of PSII photochemistry	$(F_m - F_o)/F_m$
Φ_{PSII}	Effective quantum yield of PSII photochemistry	$(F_m' - F_s)/F_m'$
Φ_{NPQ}	Quantum yield of regulated non-photochemical energy loss in PSII	$F_s/F_m' - F_s/F_m$
Φ_{NO}	Quantum yield of nonregulated energy loss in PSII	F_s/F_m
F_v'/F_m'	Efficiency of open PSII centers	$(F_m' - F_o')/F_m'$
F_v/F_o	Efficiency of the oxygen evolving complex (OEC) on the donor side of PSII	$(F_m - F_o)/F_o$
ETR	Electron transport rate	Φ_{PSII} × PAR × c × abs, where PAR is the photosynthetically active radiation, c is 0.5, and abs is the total light absorption of the leaf taken as 0.84
q_p	Photochemical quenching, representing the fraction of PSII reaction centers in open state (puddle model)	$(F_m' - F_s)/(F_m' - F_o')$
NPQ	Non-photochemical quenching reflecting the dissipation of excitation energy as heat	$(F_m - F_m')/F_m'$
EXC	Excess excitation energy	$(F_v/F_m - \Phi_{PSII})/F_v/F_m$
$1\text{-}q_L$	The fraction of PSII reaction centers in closed state (based on a “lake” model for the photosynthetic unit)	$q_p \times F_o'/F_s$

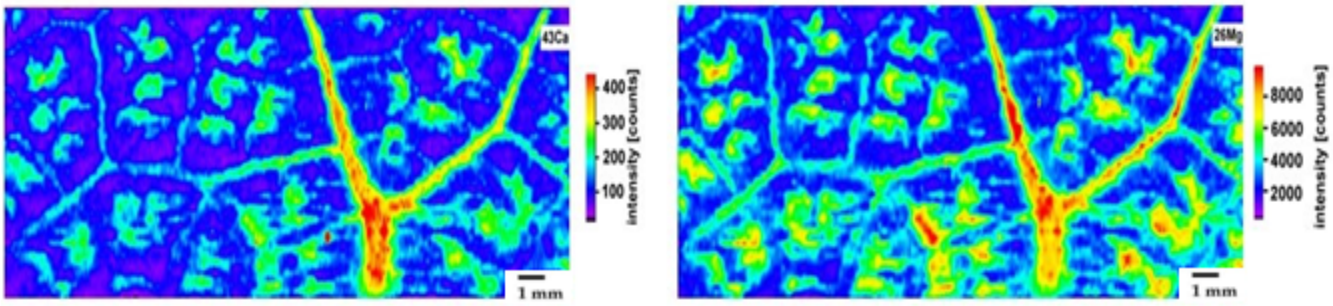

(**a**) (**b**)

Figure S1. The distribution pattern of Ca (**a**) and Mg (**b**) in a control *Salvia sclarea* leaf segment. Ca^{43} and Mg^{26} intensities were normalized using C^{13}. Scale bar: 1 mm.

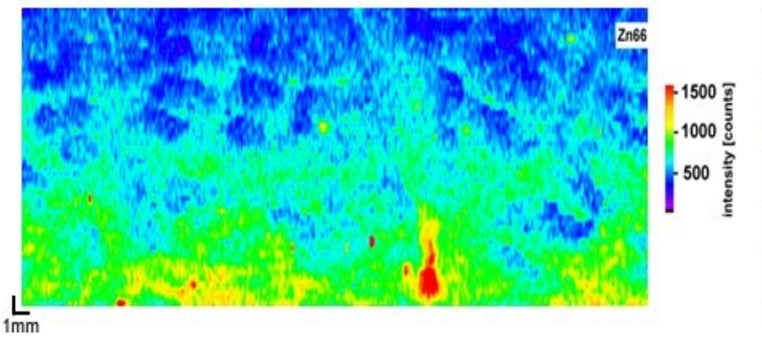

Figure S2. The distribution pattern of Zn in a control *Salvia sclarea* leaf segment. Zn^{66} intensities were normalized using C^{13}. Scale bar: 1 mm.

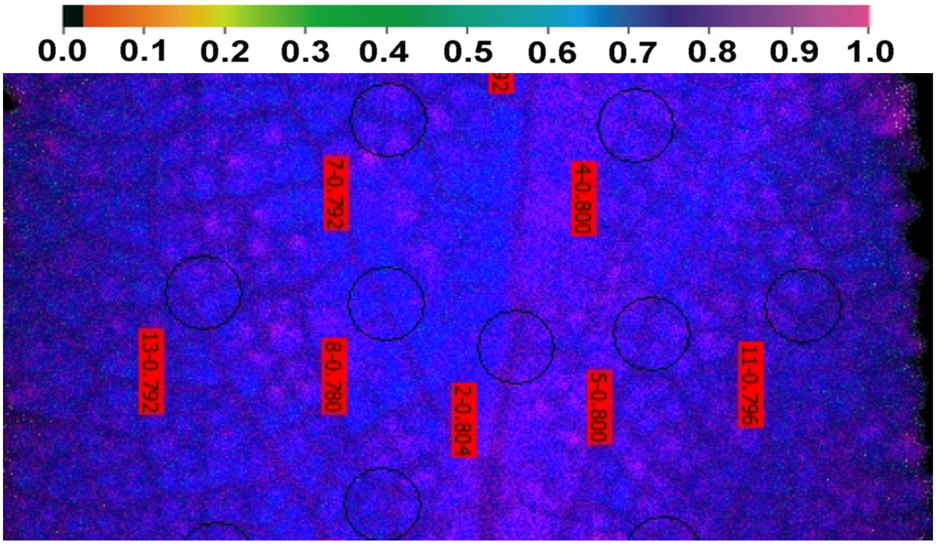

Figure S3. A color-coded leaf segment of a control *Salvia sclarea* plant showing the areas of interest (AOI) in circles together with red labels with the values of the fraction of open PSII reaction centers (q*p*) in the main leaf vein area and the intercostal fields.

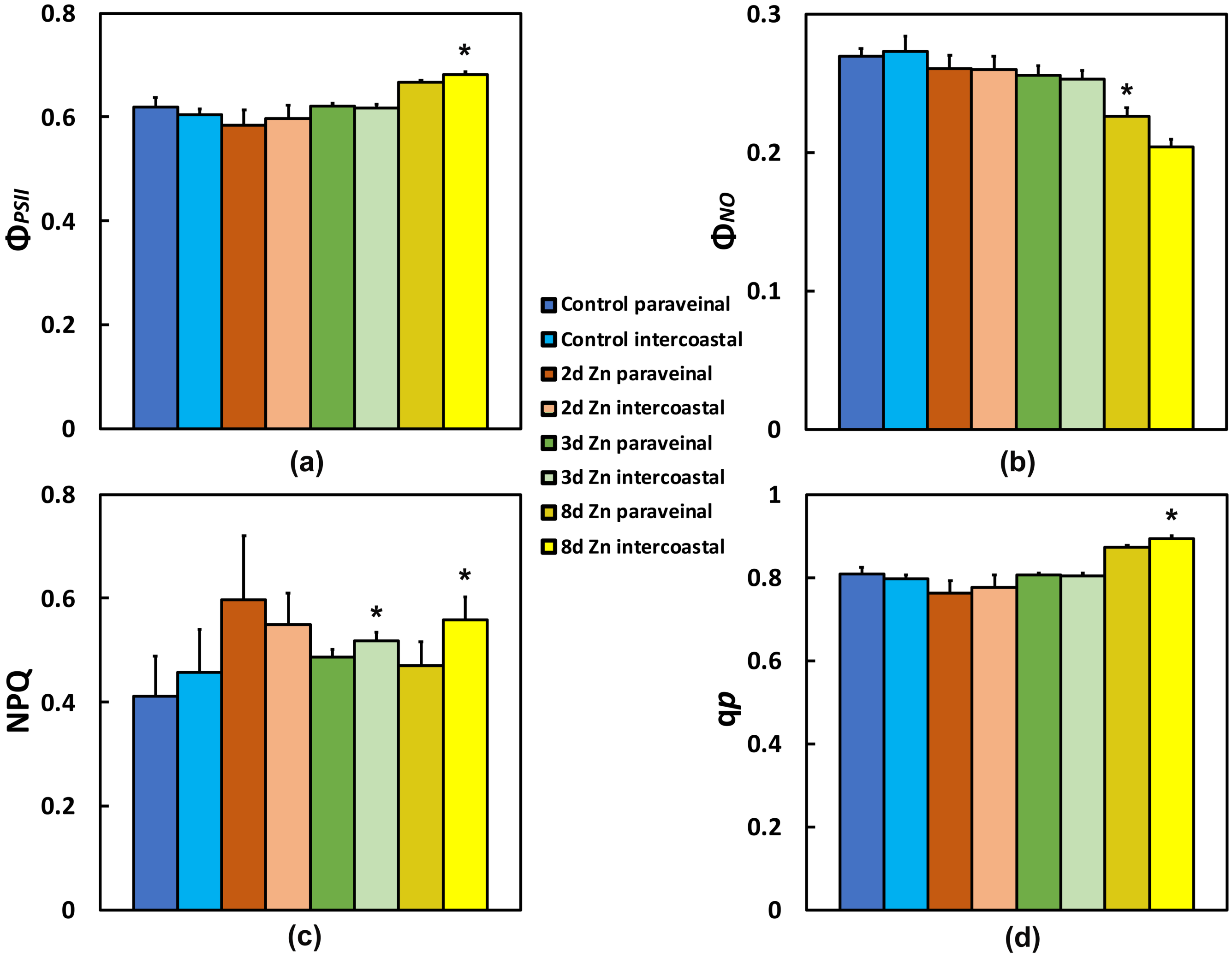

Figure S4. The effective quantum yield of PSII photochemistry (Φ_{PSII}) (**a**); the quantum yield of non-regulated energy dissipated in PSII (Φ_{NO}) (**b**); the non-photochemical quenching that reflects heat dissipation of excitation energy (NPQ) (**c**); and the fraction of open PSII reaction centers (q*p*), a measure of the redox state of quinone A (Q_A) (**d**), of the mid-vein-paraveinal areas of interest (AOI) and the intercostal field AOI, estimated at 220 μmol photons m^{-2} s^{-1} actinic light (AL) intensity, after exposure of clary sage plants to 0 (Control), 2, 3, and 8-days 900 μM Zn. An asterisk indicates significant difference at ($p < 0.05$) between mid-vein areas and intercostal field for the same day treatment. For the statistically significant differences ($p < 0.05$) a Welch's test for each independent variable between the mid vein paraveinal AOI and the intercostal AOI was conducted.

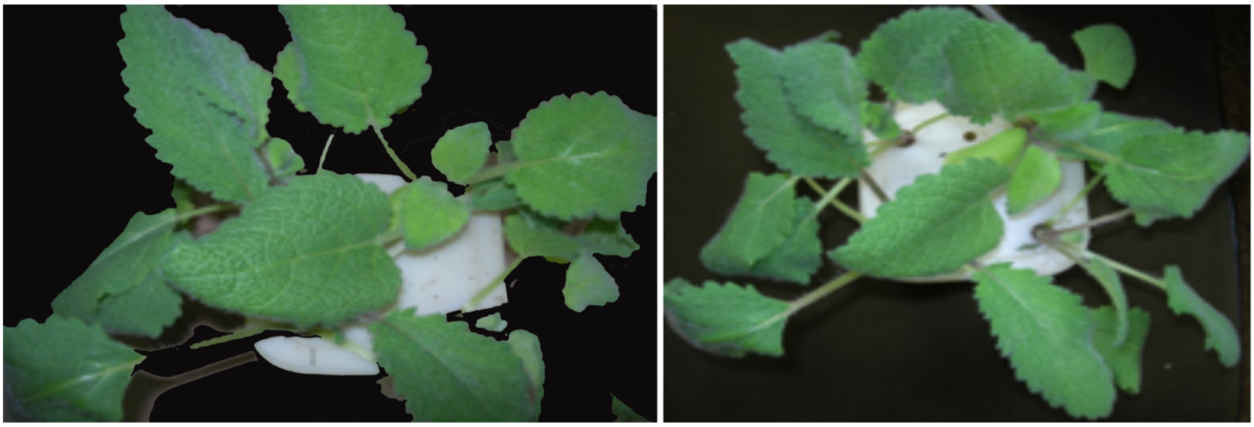

(**a**) (**b**)

Figure S5. *Salvia sclarea* L. (clary sage) plants after 8 days in Hoagland nutrient solution with 5 μM Zn, control **(a)** and after 8 days in Hoagland nutrient solution plus 900 μM Zn **(b).**
